# The current status of gene expression profilings in COVID‐19 patients

**DOI:** 10.1002/ctd2.104

**Published:** 2022-07-17

**Authors:** Mirolyuba Ilieva, Max Tschaikowski, Andrea Vandin, Shizuka Uchida

**Affiliations:** ^1^ Center for RNA Medicine, Department of Clinical Medicine Aalborg University Copenhagen Denmark; ^2^ Department of Computer Science Aalborg University Aalborg Denmark; ^3^ Institute of Economics and EMbeDS Sant'Anna School of Advanced Studies Pisa Italy; ^4^ Department of Applied Mathematics and Computer Science Technical University of Denmark Kongens Lyngby Denmark

**Keywords:** biomarker, COVID‐19, gene expression, RNA‐seq

## Abstract

**Background:**

The global pandemic of coronavirus disease 2019 (COVID‐19) caused by severe acute respiratory syndrome coronavirus 2 (SARS‐CoV‐2) has swept through every part of the world. Because of its impact, international efforts have been underway to identify the variants of SARS‐CoV‐2 by genome sequencing and to understand the gene expression changes in COVID‐19 patients compared to healthy donors using RNA sequencing (RNA‐seq) assay. Within the last two and half years since the emergence of SARS‐CoV‐2, a large number of OMICS data of COVID‐19 patients have accumulated. Yet, we are still far from understanding the disease mechanism. Further, many people suffer from long‐term effects of COVID‐19; calling for a more systematic way to data mine the generated OMICS data, especially RNA‐seq data.

**Methods:**

By searching gene expression omnibus (GEO) using the key terms, COVID‐19 and RNA‐seq, 108 GEO entries were identified. Each of these studies was manually examined to categorize the studies into bulk or single‐cell RNA‐seq (scRNA‐seq) followed by an inspection of their original articles.

**Results:**

The currently available RNA‐seq data were generated from various types of patients’ samples, and COVID‐19 related sample materials have been sequenced at the level of RNA, including whole blood, different components of blood [e.g., plasma, peripheral blood mononuclear cells (PBMCs), leukocytes, lymphocytes, monocytes, T cells], nasal swabs, and autopsy samples (e.g., lung, heart, liver, kidney). Of these, RNA‐seq studies using whole blood, PBMCs, nasal swabs and autopsy/biopsy samples were reviewed to highlight the major findings from RNA‐seq data analysis.

**Conclusions:**

Based on the bulk and scRNA‐seq data analysis, severe COVID‐19 patients display shifts in cell populations, especially those of leukocytes and monocytes, possibly leading to cytokine storms and immune silence. These RNA‐seq data form the foundation for further gene expression analysis using samples from individuals suffering from long COVID.

## 1 INTRODUCTION

The rise of next‐generation sequencing, especially RNA sequencing (RNA‐seq) has revolutionized the way we conduct research. Due to the decreased costs of performing RNA‐seq experiments, it is now commonly used as the first step of research to profile gene expression changes of one condition compared to another. Through the development of a more elaborate assay, gene expression profiling at the single‐cell level is possible, which is collectively called single‐cell RNA‐seq (scRNA‐seq). Instead, the term bulk RNA‐seq is used for RNA‐seq assay other than scRNA‐seq. It is now a common practice and requirement for most journals to deposit the generated RNA‐seq data before the publication of each study in a journal. These data are readily available from public domains, such as gene expression omnibus (GEO), ArrayExpress, and Sequence Read Archive (SRA). Such data sharing allows for secondary analysis of the previously published RNA‐seq data to discover gene expression changes from a different perspective than originally intended by combining two or more similar studies.

Severe acute respiratory syndrome coronavirus 2 (SARS‐CoV‐2) is the causative virus for the global pandemic, coronavirus disease 2019 (COVID‐19). Because of its global impact, numerous approaches, especially those using high‐throughput OMICS techniques, have been taken to characterise the genomic mutations of this virus as well as the impact on the COVID‐19 patients, especially using RNA‐seq assay. Due to the rapid mutations of RNA viruses, SARS‐CoV‐2 has mutated by acquiring more aggressive infection rates in humans.^1^ These mutations are closely monitored by performing genomic sequencing of COVID‐19 patients around the world. Although various mutations and dominant variants of SARS‐CoV‐2 have been identified, the symptoms and severity of COVID‐19 patients vary significantly depending, in part, on underlying conditions (e.g., older ages, diabetes, obesity, gender).^2^ The symptoms of COVID‐19 are diverse, especially those suffering from long‐term symptoms. The specific term, long COVID, has been developed to describe those suffering for more than 3 months.^3^ The current findings indicate that the damage to endothelial and nerve cells might be responsible for the short‐ and long‐term COVID symptoms, which result in damage to the lungs, heart, brain, and other vital organs of those infected.^4^, ^5^ With the appearance of the less life‐threatening variant of SARS‐CoV‐2, the BA.2 variant (so‐called stealth omicron^6^), it is clear that the research has shifted to understanding the chronic complications of COVID‐19, including chest pain, cough, fatigue, headaches, joint pain, loss of smell or tastes, and shortness of breath. Due to the global interest to elucidate the disease mechanism, many data have been collected, including OMICS data. Yet, one is still far from understanding the whole spectrum of the negative impact of SARS‐CoV‐2 on human health as in the case of possible causative contribution to the rise of mysterious hepatitis in children in recent weeks.^7^ Thus, it is clear that more systematic approaches are urgently needed to understand the impact of long COVID. To facilitate such approaches, this Mini‐Review surveys the current status of gene expression profilings of COVID‐19 patients using the RNA‐seq technique.

## 2 PUBLICLY AVAILABLE RNA‐SEQ DATA OF COVID‐19 PATIENTS AND COVID‐RELATED RESEARCH

To screen for genes affected by SARS‐CoV‐2 and possibly responsible for the symptoms of COVID‐19 patients, both bulk RNA‐seq and scRNA‐seq techniques have been used. Because of the global impact of COVID‐19, various types of patients’ samples and COVID‐19 related sample materials have been sequenced at the level of RNA, including whole blood, different components of blood [e.g., plasma, peripheral blood mononuclear cells (PBMCs), leukocytes, lymphocytes, monocytes, T cells], nasal swabs, and autopsy samples (e.g., lung, heart, liver, kidney) (Table 1).

**TABLE 1:** List of RNA‐seq data available from GEO. PMID stands for PubMed ID

**GEO Accession ID**	**Target cells/tissues**	**Conditions**	**Number of samples**	**Type of sequencing**	**Publication**
GSE147975	human pluripotent stem cell‐derived colonic organoids	infected with SARS‐CoV‐2 pseudo‐entry virus	2	Single‐Cell RNA‐seq	PMID: 33116299
GSE149689	peripheral blood mononuclear cells (PBMCs)	healthy donors, flu, or COVID‐19 patients	20	Single‐Cell RNA‐seq	PMID: 32651212
GSE149973	Vero 6 or Calu 3 cells	infected with BavPat1/2020 EPI_ISL_406862	26	Bulk RNA‐seq, ribosome‐profiling	PMID: 32906143
GSE150316	lung, jejunum, heart, liver, kidney, bowel, fat, skin, bone marrow, placenta	autopsy samples from patients deceased due to SARS‐Cov2 infection	88	Bulk RNA‐seq	PMID: 33298930
GSE150392	human induced pluripotent stem cell‐derived cardiomyocytes	infected with SARS‐CoV‐2	6	Bulk RNA‐seq	PMID: 32835305, 33805011
GSE150819	human bronchial organoids, primary human bronchial epithelial cells, or A549 cell	infected with SARS‐CoV‐2 in the presence of absence of camostat	18	Bulk RNA‐seq	*https://www.biorxiv.org/content/10.1101/2020.05.25.115600v2.article‐info*
GSE150861	peripheral blood mononuclear cells (PBMCs)	severe COVID‐19 patients treated with Tocilizumab (time‐course)	7	Single‐Cell RNA‐seq	*^PMID: 32764665^*
GSE151161	whole blood	COVID‐19 patients treated with abatacept (time‐course)	76	Bulk RNA‐seq	*^PMID: 34075090^*
GSE151878	human embryonic stem cell‐derived cardiomyocytes	infected with SARS‐CoV‐2 Pseudo‐entry virus and co‐cultured with macrophages	3	Single‐Cell RNA‐seq	*^PMID: 33236003^*
GSE151973	olfactory epithelium, nasal respiratory epithelium	COVID‐19 patients	6	Bulk RNA‐seq	*^PMID: 33251489^*
GSE152522	virus‐reactive memory CD4+ T cells	healthy donors or COVID‐19 patients	78	Single‐Cell RNA‐seq, TCR‐seq	*^PMID: 33096020^*
GSE152641	whole blood	healthy donors or COVID‐19 patients	86	Bulk RNA‐seq	*^PMID: 33437935^*
GSE153931	virus‐reactive memory CD8+ T cells	healthy donors or COVID‐19 patients	45	Single‐cell RNA‐seq	*^PMID: 33478949^*
GSE154244	nasopharyngeal swab	COVID‐19 patients	4	Bulk RNA‐seq	*^PMID: 33413422^*
GSE154311	neutrophils (CD16 subtypes)	severe COVID‐19 patients	9	Bulk RNA‐seq	*^PMID: 33986193^*
GSE154567	blood buffy coat	COVID‐19 patients	9	Single‐cell RNA‐seq	*^PMID: 32743611, 33357411^*
GSE155223	peripheral blood mononuclear cells (PBMCs)	severe COVID‐19 patients (time‐course)	18	single‐cell RNA‐seq	*^PMID: 35064122^*
GSE155249	macrophages and T cells	bronchoalveolar lavage fluid from COVID‐19 positive, COVID‐19 negative with bacterial pneumonia secondary to infection with Pseudomonas aeruginosa and Acinetobacter baumannii, COVID‐19 negative, intubated for airway protection to facilitate endoscopy for severe gastrointestinal bleeding without pneumonia	19	Bulk RNA‐seq	*^PMID: 33429418^*
GSE155286	lung organoid	human lung‐only mice (LoM) infected with recombinant coronaviruses SARS‐CoV, MERS‐CoV, SARS‐CoV‐2, full length bat coronaviruses WIV1 or SHC014	13	Bulk RNA‐seq	*^PMID: 33561864^*
GSE155518	AT2 cells	cultured in 3D and infected with SARS‐CoV2	6	Bulk RNA‐seq	*^No^*
GSE157103	leukocytes	healthy donors or COVID‐19 patients	126	Bulk RNA‐seq	*^PMID: 33096026^*
GSE157344	blood or bronchoalveloar lavage	healthy donors or COVID‐19 patients	54	Single‐Cell RNA‐seq	*^PMID: 33674591^*
GSE157403	kidney	COVID‐19 patient	1	Bulk RNA‐seq	*^PMID: 33942030^*
GSE157490	Calu‐3 cells	infected with SARS‐CoV‐2 (time‐course)	127	Bulk RNA‐seq, RPF‐seq, QTI‐seq, sRNA‐seq	*^PMID: 34433827^*
GSE157789	leukocytes and lymphocytes	healthy donors, severe COVID‐19, or bacterial acute respiratory distress syndrome patients with or without dexamethasone treatment	31	Single‐Cell RNA‐seq	*^PMID: 34782790^*
GSE157852	choroid plexus organoids	infected with SARS‐CoV‐2 (time‐course)	9	Bulk RNA‐seq	*^PMID: 33010822^*
GSE158127	Lung	healthy donors or patients with prolonged COVID‐19	22	Single‐cell RNA‐seq	*^PMID: 33257409^*
GSE159556	primary human pancreatic islet cells	infected with SARS‐CoV‐2	5	Single‐cell RNA‐seq	*^PMID: 34081913^*
GSE159678	monocytes	COVID‐19 patients and treated with hydroxychloroquine in vitro	47	RNA‐seq, ChIP‐seq	*^PMID: 33377122^*
GSE160351	peripheral monocytes	healthy donors or COVID‐19 patients	9	Bulk RNA‐seq	*^PMID: 33208929, 34145258^*
GSE161225	Skin	healthy controls, maculopapular drug rash with or without COVID‐19 infection	15	Bulk RNA‐seq	*^PMID: 34157151^*
GSE162316	A549	stably expressing ACE2 and treated with CoV2‐miR‐7a.1 and CoV2‐miR‐7a.2, or control mimic RNA	16	small RNA‐seq	*^PMID: 34914162^*
GSE162323	Calu‐3 cells	infected with SARS‐CoV‐2 (time‐course)	42	Bulk RNA‐seq, ribosome profiling	*^PMID: 33979833^*
GSE162562	peripheral blood mononuclear cells (PBMCs)	healthy donors, asymptomatic COVID‐19 patients, highly exposed seronegative subjects, non‐Ischgl community (ski resort in Austria) COVID‐19 patients with mild symtoms, or highly exposed seronegative non‐Ischgl community subjects	108	Bulk RNA‐seq	*^PMID: 33608566, 34100027^*
GSE162629	Caco‐2 cells	infected with SARS‐CoV‐2 GFP delN P1 or P10 virus	2	Bulk RNA‐seq	*^No^*
GSE162911	lung, trachea, heart	regions of interest (ROIs) from FFPE samples of 9 COVID‐19 patients	784	Bulk RNA‐seq	*^PMID: 33915569^*
GSE163005	cerebrospinal fluid‐derived leukocytes	Neuro‐COVID, non‐inflammatory or autoimmune neurological diseases, or viral encephalitis	38	Single‐cell RNA‐seq	*^PMID: 33382973^*
GSE163151	blood or nasopharyngeal swab	healthy donors, individuals with SARS‐CoV‐2 infection, other viral acute respiratory infections, non‐viral acute respiratory illness	404	Bulk RNA‐seq	*^PMID: 33536218^*
GSE164013	Lung	80 regions of interest (ROIs) from autopsy FFPE lung tissues from a cohort of 5 patients with positive SARS‐CoV‐2 nasopharyngeal swab on admission	80	Bulk RNA‐seq	*^PMID: 33915569^*
GSE164332	brain (frontal cortex)	healthy donors or COVID‐19 patients	16	Bulk RNA‐seq	*^PMID: 34022369^*
GSE164948	peripheral blood mononuclear cells (PBMCs)	healthy donors, COVID‐19 or community‐acquired pneumonia patients	4	Single‐cell RNA‐seq	*^PMID: 34424199^*
GSE165080	peripheral blood mononuclear cells (PBMCs)	healthy donors or COVID‐19 patients	53	Single‐cell RNA‐seq	*^PMID: 35281000^*
GSE165193	umbilical cord blood mononuclear cells	infants born to mothers infected with SARS‐CoV‐2 in the third trimester	12	Single‐cell RNA‐seq	*^PMID: 33758834, 34750520^*
GSE166530	nasopharyngeal or oropharyngeal swabs	healthy donors or COVID‐19 patients	41	Bulk RNA‐seq	*^PMID: 34586723^*
GSE166990	human induced pluripotent cells	overexpression of ACE2 and infected with SARS‐CoV‐2	6	Bulk RNA‐seq	*^PMID: 33880436^*
GSE166992	peripheral blood mononuclear cells (PBMCs)	healthy donors or COVID‐19 patients	9	Single‐cell RNA‐seq	*^PMID: 33691089^*
GSE167075	Caco‐2 cells	infected with SARS‐CoV‐2 and treated with shRNA against control sequence or m6A writer, METTL3	16	Bulk RNA‐seq	*^PMID: 33961823^*
GSE167747	induced pluripotent stem cell‐derived human kidney organoids/kidney autopsy	infected with SARS‐CoV‐2/COVID‐19 patients	6	Single‐cell RNA‐seq	*^PMID: 35032430^*
GSE167930	peripheral blood mononuclear cells (PBMCs)	healthy donors or COVID‐19 patients	40	Bulk RNA‐seq	*^PMID: 34586734^*
GSE168215	bronchial brushing	COVID‐19 patients	9	Bulk RNA‐seq	*^PMID: 34937051^*
GSE168797	A549 cells	overexpression of ACE2	24	Bulk RNA‐seq	*^PMID: 33758843^*
GSE169241	hearts/human embryonic stem cell‐derived cardiomyocytes	healthy donors or COVID‐19 patients/infected with SARS‐CoV‐2 and treated with Ranolazine or Tofacitinib	23	Bulk RNA‐seq	*^PMID: 33853355^*
GSE171110	whole blood	healthy donors or COVID‐19 patients	54	Bulk RNA‐seq	*^PMID: 34127958^*
GSE171370	human pluripotent stem cell‐derived cardiomyocytes (hPSC‐CMs)	overexpression of Orf9c, a SARS‐CoV‐2 encoded gene	6	Bulk RNA‐seq	*^PMID: 35180394^*
GSE171381	decidua or placental villi	pregnant women with and without COVID‐19	9	Single‐cell RNA‐seq	*^PMID: 33969332^*
GSE171555	peripheral blood mononuclear cells (PBMCs)	healthy donors, COVID‐19 inpatients (hospitalized) and outpatients (infected), or uninfected close contacts (exposed)	48	Single‐cell RNA‐seq	*^PMID: 33870241^*
GSE171668	lung, heart, liver, kidney	severe COVID‐19 patients	188	Bulk RNA‐seq, single‐cell RNA‐seq, single‐nucleus RNA‐seq	*^PMID: 33915569^*
GSE172114	whole blood	critical and non‐critical COVID‐19 patients at hospitalization	69	Bulk RNA‐seq	*^PMID: 34698500^*
GSE173507	Vero E6, A549‐ACE2, or BEAS‐2B cells	infected with SARS‐CoV‐2	4	Bulk RNA‐seq	*^PMID: 35233578; 35313591^*
GSE174083	whole blood	four‐time points before and after the meditation retreat	388	Bulk RNA‐seq	*^PMID: 34907015^*
GSE174668	A549 or HepG2 cells	incubated with extracellular vesicles (EVs) isolated from healthy donors, presymptomatic S1, hyperinflammatory S2, convalescent S3, or resolution S4 phases of COVID‐19 patients	30	Bulk RNA‐seq	*^PMID: 34158670^*
GSE174745	brain (ventral midbrain)	non‐COVID‐19 or COVID‐19 patients	15	Bulk RNA‐seq	*^No^*
GSE176201	peripheral blood mononuclear cells (PBMCs) or BAL T cells	healthy donors or aged COVID‐19 convalescents	14	Single‐cell RNA‐seq, TCR‐seq	*^PMID: 34591653^*
GSE176269	nasal wash cells	adults with COVID‐19, influenza A, or no disease (healthy)	116	Single‐cell RNA‐seq	*^No^*
GSE176479	cardiac microvascular endothelial cells	exposed to platelet releasate originating from patients with COVID‐19 or healthy controls	14	Bulk RNA‐seq	*^PMID: 34516880^*
GSE176498	plasma	healthy controls, non‐severe or severe COVID‐19 patients	47	Bulk RNA‐seq	*^PMID: 34755842^*
GSE178331	pooled human umbilical vein endothelial cells (pHUVECs)/blood	pHUVECs were stimulated with bloods from COVID‐19 negative, mild, moderate, or severe patients	25	Bulk RNA‐seq	*^PMID: 35405523^*
GSE178824	granulocytic‐myeloid derived suppressor cells	healthy donors, severe, asymptomatic, or convalescent COVID‐19 patients	16	Bulk RNA‐seq	*^PMID: 34341659^*
GSE179448	T regulatory cells	healthy donors or COVID‐19 patients	86	Bulk RNA‐seq	*^PMID: 34433692^*
GSE181238	placenta	healthy controls, COVID‐19+ mothers, or mothers with on‐COVID related inflammatory pathologies	31	Bulk RNA‐seq	*^No^*
GSE182297	oral pharynx, prefrontal cortex, nasal pharynx, olfactory bulb, salivary gland, tongue, heart, liver, lung, or kidney	one COVID‐19 patient compared to a pool of brain RNA from multiple donors as control	22	Bulk RNA‐seq	*^PMID: 34506752^*
GSE182917	liver, heart, kidney, spleen, lung	healthy control donors or SARS‐CoV‐2 infected patients	24	Bulk RNA‐seq	*^PMID: 35022412^*
GSE183716	peripheral blood mononuclear cells (PBMCs)	multisystem inflammatory syndrome in children (MIS‐C) after SARS‐CoV‐2 infection	8	Single‐cell RNA‐seq, CITE‐seq	*^No^*
GSE187420	Calu‐3 cells	control, SARS‐CoV‐2 infection, or IMD‐0354 treatment followed by SARS‐CoV‐2 infection	9	Bulk RNA‐seq	*^No^*
GSE188847	brain (frontal cortex)	severe COVID‐19 or unaffected patients	24	Bulk RNA‐seq	*^No^*
GSE189039	peripheral blood mononuclear cells (PBMCs)	COVID‐19 patients infected by SARS‐CoV‐2 Beta varient (Beta) or SARS‐CoV‐2 naïve vaccinated individuals	40	Bulk RNA‐seq	*^PMID: 35465056^*
GSE189506	Serum	COVID‐19 patients (6 survivors, 6 deceased) with multifocal interstitial pneumonia and requiring oxygen therapy	12	small RNA‐seq	*^PMID: 35122770^*
GSE190193	lung epithelial cells derived from human induced pluripotent stem cells (hiPSC)	infected with SARS‐CoV‐2	9	Bulk RNA‐seq	*^No^*
GSE190680	buffy coat	COVID‐19 patients infected by SARS‐CoV‐2 Alpha varient with or without the escape mutation	100	Bulk RNA‐seq	*^PMID: 35181735^*
GSE190747	peripheral blood mononuclear cells (PBMCs)	recovered COVID‐19 patients or naïve individuals who had received the BNT162b mRNA vaccine	115	Bulk RNA‐seq	*^No^*
GSE192391	peripheral blood mononuclear cells (PBMCs)	COVID‐19 patients (time‐course)	30	Single‐cell RNA‐seq	*^PMID: 35169146^*
GSE193722	hamster hearts or human embryonic stem cell–derived sinoatrial node‐like pacemaker cells	infected with SARS‐CoV‐2	21	Bulk RNA‐seq	*^PMID: 35255712^*
GSE193770	T cells	healthy controls, multiple sclerosis patients or COVID‐19 patients	10	Single‐cell RNA‐seq	*^PMID: 35258337^*
GSE196455	monocytes	male and female donors treated with mock, ORF8, or hIL‐17A	12	Bulk RNA‐seq	*^PMID: 35343786^*
GSE197204	whole blood	critically‐ill COVID‐19 patients obtained at admission in an Intensive Care Unit	56	Bulk RNA‐seq	*^No^*
GSE198256	monocytes	healthy controls, acute or convalescent COVID‐19 patients	34	Bulk RNA‐seq	*^No^*
GSE198449	whole blood	COVID‐19 Health Action Response for Marines (CHARM) study: samples collected from 475 subjects at different time points as part of SARS‐Cov‐2 initial outbreak and later surveillance on the Marine recruits	1858	Bulk RNA‐seq	*^PMID: 35479098^*
GSE199272	humanized mice (MISTRG6‐hACE2) infected with SARS‐CoV‐2	lung	3	Single‐cell RNA‐seq	*^No^*
GSE200561	humanized mice (MISTRG6‐hACE2) infected with SARS‐CoV‐2	lung	16	Bulk RNA‐seq	*^No^*

### 2.1 Whole blood

The drawing of blood is a standard medical practice to diagnose various diseases. Thus, it is no surprise that many RNA‐seq data of whole blood of COVID‐19 patients compared to that of healthy donors are available. For example, the analysis of RNA‐seq data of whole blood from 42 severe hospitalized COVID‐19 patients compared to 10 healthy donors shows that 4 079 genes are differentially expressed at the threshold of 1.5‐fold change.^8^ Not surprisingly, many genes involved in immune response (e.g., neutrophil and interferon signalling, T and B cell receptor responses) are differentially regulated, especially *CD177*, a marker of neutrophil activation. Another study comparing RNA‐seq data of 46 critical (in the intensive care unit under mechanical ventilation) and 23 non‐critical COVID‐19 patients shows that genes involved in inflammatory response, myeloid cell activation, and neutrophil degranulation are enriched in critical COVID‐19 patients, especially the metalloprotease *ADAM9*.^9^

**FIGURE 1:**
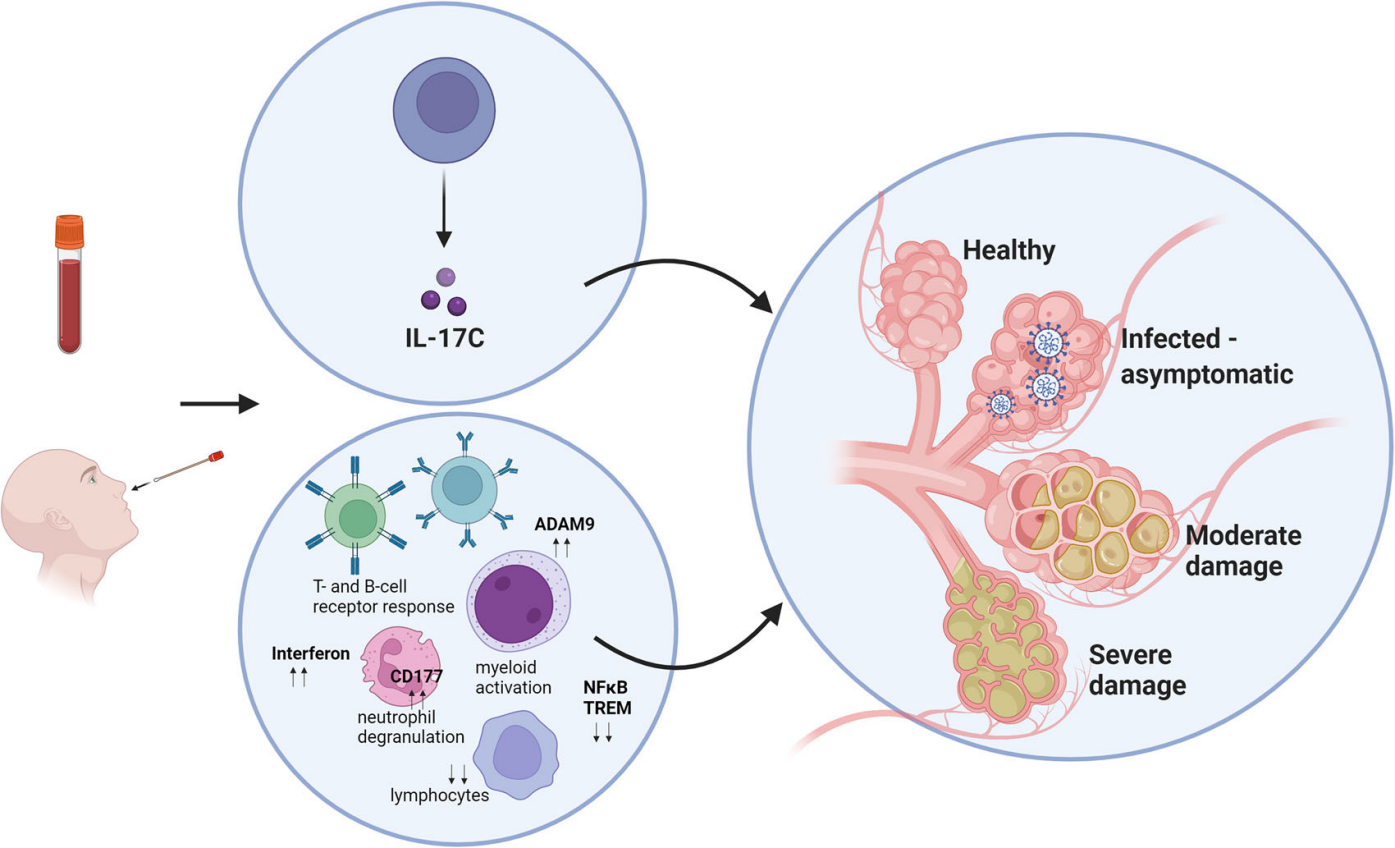
RNA‐seq data of whole blood and nasal swabs of COVID‐19 patients compared to healthy donors. Genes involved in immune response (e.g., neutrophil and myeloid activation, T‐ and B‐cell response, interferon signalling) are enriched in critical COVID patients. *CD177* and the metalloprotease *ADAM9* are upregulated, while *NFkB*, *TREM1*, and lymphocyte‐related genes are down‐regulated in severe COVID‐19 patients compared to the healthy donors, suggesting an overall dysregulated immune response. In contrast, asymptomatic infected patients show elevated level of serum IL‐17C. Created with BioRender.com

Compared to people with underlying conditions, many people infected with SARS‐CoV‐2 are asymptomatic. Thus, RNA‐seq data of COVID‐19 Health Action Response for Marines (CHARM) study is of great interest because it collected whole blood from 475 subjects at different time points during SARS‐Cov‐2 initial outbreak and later surveillance on the United States Marine recruits.^10^ Yet, the original publication of this study did not explore the RNA‐seq data in detail. This is because the study concentrated more on proteomic analysis, which identified the elevated level of serum IL‐17C in asymptomatic participants compared to those with COVID‐19 symptoms (Figure 1). As this study generated time‐course 1 858 RNA‐seq data, re‐analysis of RNA‐seq data will be of great interest to further elucidate the gene expression changes associated with COVID‐19 symptoms.

### 2.2 Peripheral blood mononuclear cells

Besides whole blood, different components of blood were used to perform RNA‐seq assay. A PBMC is any blood cell having round nucleus, including lymphocytes [T cells, B cells, natural killer (NK) cells] and monocytes.^11^, ^12^ Because PMBCs include different immune cell types, scRNA‐seq assay is employed to decipher transcriptome dynamics and cell‐type differences in COVID‐19 patients compared to healthy donors. For example, the analysis of scRNA‐seq data of PBMCs collected from 11 healthy donors, 5 asymptomatic individuals, 33 individuals with moderate COVID‐19 symptoms, 10 individuals with severe COVID‐19 symptoms, and two time‐point data of two individuals with severe COVID‐19 symptoms identified 76 cell subpopulations associated with various clinical presentations of COVID‐19 patients,^13^ highlighting the complicated cell‐type landscapes of COVID‐19 symptoms. Although such identification of cell subpopulations is important, further follow‐up studies focusing on the functionalities of these subpopulations of cells are necessary.

**FIGURE 2:**
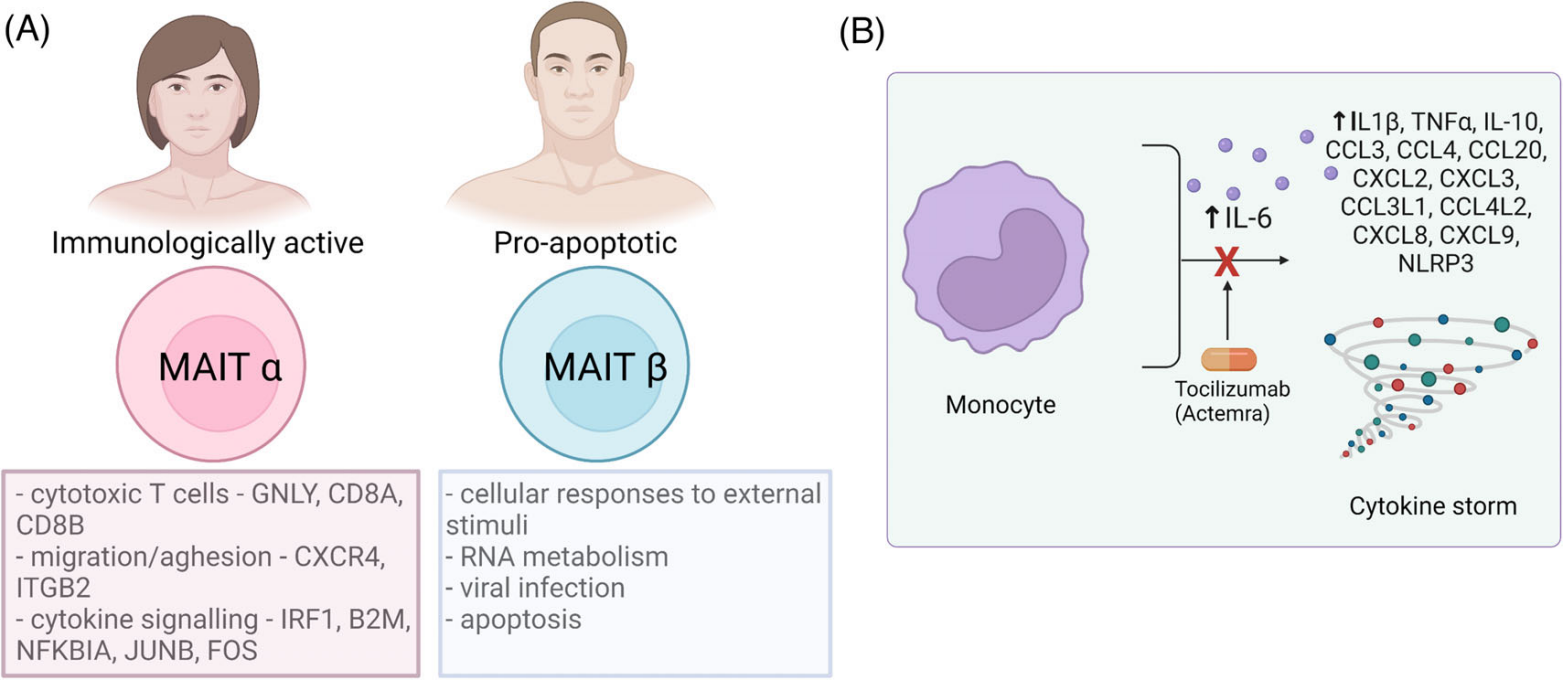
Immune profilings of COVID‐19 patients. (A) Mucosal‐associated invariant T (MAIT) cells are identified in two subpopulations: MAITα and MAITß. MAITα are immunologically active and higher in frequency in female, while MAITß are pro‐apoptotic and are dominant in male. Female‐specific MAITα cells have a protective effect, possibly related to reduced mortality rate and complications in females compared to males. (B) scRNA‐seq after Tocilizumab (Actemra) treatment of severe COVID‐19 patients. Tocilizumab targets IL6 and thus suppresses the cytokine storm caused by monocyte subpopulation of severe COVID‐19 patients. Created with BioRender.com

Across all age groups, males have a higher rate of respiratory intubation, a longer length of hospital stay, and a higher death rate from COVID‐19 compared to females.^14^ To address the gender differences in COVID‐19 patients, scRNA‐seq combined with flow cytometry analysis of 10 healthy donors, 9 COVID‐19 inpatients (hospitalized), 19 outpatients (infected), and 7 uninfected close contacts (exposed) show that circulating mucosal‐associated invariant T (MAIT) cells were recruited to airway tissues more robustly in female COVID‐19 patients compared to male COVID‐19 patients as circulating MAIT cells are higher in frequencies in females than males in the healthy setting.^15^ Interestingly, this study identified two subpopulations of MAIT cells, MAITα and MAITβ (Figure 2A). The authors defined MAITα cells to be immunologically active based on the enriched expressions of genes associated with cytotoxic T cells (*GNLY*, *CD8A*, and *CD8B*), migration/adhesion (*CXCR4* and *ITGB2*), and cytokine signalling (*IRF1*, *B2M*, *NFKBIA*, *JUNB*, and *FOS*). In contrast, MAITβ cells are defined as pro‐apoptotic based on the enriched expressions of genes categorized under cellular responses to external stimuli, metabolism of RNA, viral infection, and programmed cell death but not immune processes. In the healthy setting, MAITα cells are dominant in females, while MAITβ cells are dominant in males. Based on these findings, the authors conclude that female‐specific protective MAIT subpopulation might be responsible for the reduced severity of COVID‐19 symptoms and death.

Although COVID‐19 vaccines have been developed to reduce the mortality rate, the effective treatment of COVID‐19 patients is still lacking. Up until now, some therapeutic approaches have been taken. One of such is the usage of Tocilizumab (Actemra), which is an immunosuppressive drug targeting IL6. Using time‐course scRNA‐seq experiment of severe COVID‐19 patients treated with Tocilizumab, it was found that a subpopulation of monocytes contributes to the inflammatory cytokine storms of severe COVID‐19 patients. This monocyte subpopulation expresses *CCL3*, *IL6*, *IL10*, *TNF*, inflammation‐related chemokine genes (*CCL4*, *CCL20*, *CXCL2*, *CXCL3*, *CCL3L1*, *CCL4L2*, *CXCL8*, and *CXCL9*), and inflammasome activation‐associated genes (*NLRP3* and *IL1B*).^16^ Further, humoral and cell‐mediated antiviral immune responses were sustained even upon treatment with Tocilizumab, suggesting that further treatment targeting these cell populations is needed for COVID‐19‐related cytokine storms (Figure 2B).

### 2.3 Nasal swabs

Nasal or nasopharyngeal swabs are a common method to test for the presence of SARS‐CoV‐2. Besides detecting fragments of viral RNA, genome‐wide transcriptomic analysis of the host (i.e., COVID‐19 patients) can be performed. For example, by comparing RNA‐seq data generated from naso/oropharyngeal swabs of 36 COVID‐19 Indian patients hospitalized during the first surge of COVID‐19 to those of 5 COVID‐19 negative control samples, 251 up‐ and 9 068 down‐regulated genes were identified at the threshold of two‐fold changes and adjusted *p*‐value < .05.^17^ The differentially expressed genes include up‐regulation of genes involved in innate immune response (e.g., interferon signalling, response to virus) and down‐regulation of genes involved in membrane potentials and neurotransmitter transport as well as cardiac, muscular, and neurological processes, suggesting that significant down‐regulation of host transcriptomes can be monitored via nasal swabs.

By performing RNA‐seq assay of whole blood and/or nasopharyngeal swabs of COVID‐19 patients compared to healthy donors and individuals with other viral acute respiratory infections (i.e., influenza or seasonal coronavirus infection) or non‐viral acute respiratory illness (i.e., bacterial sepsis) (a total of 404 bulk RNA‐seq data), the activation of interferon‐mediated antiviral pathways and inhibition of other immune and inflammatory pathways (e.g., nuclear factor κB, TREM1, NK cell signalling pathways) were identified, suggesting an overall dysregulated immune response in COVID‐19 patients.^18^ This study is particularly interesting as COVID‐19 specific gene expression changes were inferred by comparing it to other infectious diseases.

### 2.4 Autopsy and biopsy samples

It is now clear that the first response to the infection of SARS‐CoV‐2 is through innate immune responses, leading to strong and dysregulated inflammatory responses and prolonged effects in various tissues.^19^ Thus, gene expression profilings of autopsy samples from COVID‐19 patients are informative in understanding the prolonged effects of SARS‐CoV‐2 on the human body. By developing a COVID‐19 autopsy biobank consisting of 11 organs and 17 donors, scRNA‐seq experiment was performed to profile 24 lungs, 16 kidneys, 16 liver and 19 heart autopsy tissues of individuals who passed away from COVID‐19.^20^ Through the detailed analysis of these data, the authors uncovered altered cellular compartments, especially in lungs, where defect in alveolar type 2 differentiation was recorded. This study provides a valuable source of autopsy samples as well as OMICS data, including bulk RNA‐seq, scRNA‐seq, and single‐nucleus RNA‐seq data. It would be of interest to compare these data to other RNA‐seq data of autopsy samples^21^, ^22^, ^23^ (Table 1) to identify common defects in tissue regeneration in COVID‐19 patients in regards to dysregulated signalling pathways.

**FIGURE 3:**
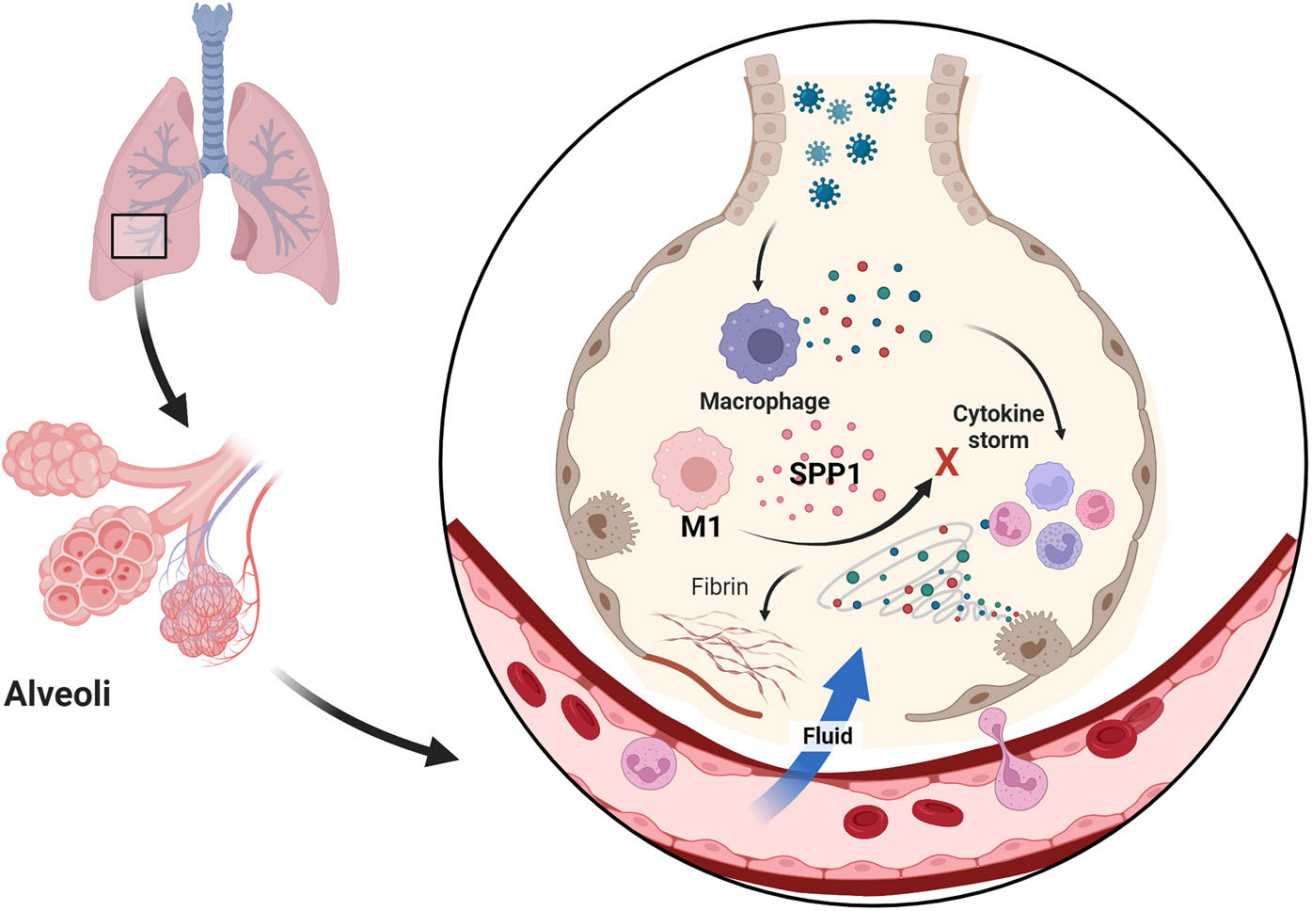
scRNA‐seq of lungs of COVID‐19 patients. Severe COVID‐19 patients have a higher level of neutrophiles and decreased level of lymphocytes. Pro‐inflammatory M1 macrophages expressing SPP1 are associated with suppression of cytokine storm and better prognosis for severe COVID‐19 patients. Created with BioRender.com

Without a doubt, the lungs are the most affected organ by SARS‐CoV‐2. Thus, intensive research focusing on gene expression profilings in lungs has been conducted. For example, scRNA‐seq data of bronchoalveolar lavage fluids (BAL) and matched peripheral blood samples from 21 severe COVID‐19 patients admitted to intensive care units (ICU) and on peripheral blood of 6 mild COVID‐19 patients and 5 healthy donors show that the severe COVID‐19 patients had a higher proportion of neutrophils and decreased proportion of lymphocytes in their blood samples compared to other two sample groups.^24^ In BAL, the gene expressions of pro‐inflammatory M1 macrophages [characterized by the expression of SPP1 (osteopontin)] were induced and associated with a better prognosis for severe COVID‐19 patients (Figure 3). Based on these data, the authors conclude that immune silence in severe COVID‐19 patients may stem from myeloid dysregulation and lymphoid impairment. Just as with any other scRNA‐seq study, further follow‐up studies with more functional and mechanistic studies of the identified subpopulations of cells are necessary to firmly establish the observations made by scRNA‐seq data analysis.

## 3 CONCLUSION

The longitudinal cohort study of COVID‐19 patients who had survived hospitalization indicates that even two years after discharge from Jin Yin‐tan Hospital (Wuhan, China), survivors with long COVID symptoms had a lower health‐related quality of life (HRQoL), worse exercise capacity, more mental health abnormality, and increased health‐care use after discharge compared to those without long COVID symptoms.^25^ This indicates that mechanistic understanding of long‐term effects of COVID‐19 is urgently needed. To this end, more RNA‐seq data should be generated from individuals with long COVID symptoms. Such newly generated data can be compared to the previously generated data as listed in Table 1 to perform a comparative analysis of transcriptomic data to understand how gene expression changes affect COVID‐19 patients. There are some studies already published that performed secondary analysis of previously generated RNA‐seq and microarray data of COVID‐19 patients compared to healthy donors and individuals with other illnesses [e.g., SARS and the Middle East respiratory syndrome (MERS), lupus].^26^, ^27^, ^28^, ^29^ Yet, to understand the disease mechanism of SARS‐CoV‐2, RNA‐seq data from long‐COVID patients should be generated not only from blood or blood‐related materials but also from tissue biopsy samples from the affected areas by SARS‐CoV‐2. Furthermore, more systematic analysis of RNA‐seq data combined with other OMICS data (e.g., genomics, proteomics, metabolomics), especially those of time‐course data, are urgently needed. These data should be analysed not only for gene expression changes but also for gene regulatory networks as well as using machine learning algorithms to train and predict the early diagnostic biomarkers of long COVID. It is also important to note that gene expression changes should be verified with protein expressions, including proteomics and fluorescence‐activated cell sorting (FACS) analysis. Such combined approaches will help to understand the disease mechanisms of SARS‐CoV‐2 causing long COVID.

## CONFLICT OF INTEREST

The authors declare that there is no conflict of interest that could be perceived as prejudicing the impartiality of the research reported.

## References

[1] Morales AC , Rice AM , Ho AT , et al. Causes and consequences of purifying selection on SARS‐CoV‐2. Genome Biol Evol. 2021;13(10):evab196. 10.1093/gbe/evab196 34427640 PMC8504154

[2] Palaiodimos L , Kokkinidis DG , Li W , et al. Severe obesity, increasing age and male sex are independently associated with worse in‐hospital outcomes, and higher in‐hospital mortality, in a cohort of patients with COVID‐19 in the Bronx, New York. Metabolism. 2020;108:154262. 10.1016/j.metabol.2020.154262 32422233 PMC7228874

[3] Goertz YMJ , Van Herck M , Delbressine JM , et al. Persistent symptoms 3 months after a SARS‐CoV‐2 infection: the post‐COVID‐19 syndrome? ERJ Open Res. 2020;6(4):00542‐2020. 10.1183/23120541.00542-2020 33257910 PMC7491255

[4] Jain U . Effect of COVID‐19 on the Organs. Cureus. 2020;12(8):e9540. 10.7759/cureus.9540 32905500 PMC7470660

[5] Iadecola C , Anrather J , Kamel H . Effects of COVID‐19 on the nervous system. Cell. 2020;183(1):16‐27 e1. 10.1016/j.cell.2020.08.028 32882182 PMC7437501

[6] Vo GV , Bagyinszky E , An SSA . COVID‐19 genetic variants and their potential impact in vaccine development. Microorganisms. 2022;10(3):10030598. 10.3390/microorganisms10030598 PMC8954257 35336173

[7] The Lancet Infectious D . Explaining the unexplained hepatitis in children. Lancet Infect Dis. 2022;22:743 10.1016/S1473-3099(22)00296-1 35569492

[8] Levy Y , Wiedemann A , Hejblum BP , et al. CD177, a specific marker of neutrophil activation, is associated with coronavirus disease 2019 severity and death. iScience. 2021;24(7):102711. 10.1016/j.isci.2021.102711 34127958 PMC8189740

[9] Carapito R , Li R , Helms J , et al. Identification of driver genes for critical forms of COVID‐19 in a deeply phenotyped young patient cohort. Sci Transl Med. 2022;14(628):eabj7521. 10.1126/scitranslmed.abj7521 34698500

[10] Soares‐Schanoski A , Sauerwald N , Goforth CW , et al. Asymptomatic SARS‐CoV‐2 infection is associated with higher levels of serum IL‐17C, matrix metalloproteinase 10 and fibroblast growth factors than mild symptomatic COVID‐19. Front Immunol. 2022;13:821730. 10.3389/fimmu.2022.821730 35479098 PMC9037090

[11] He D , Yang CX , Sahin B , et al. Whole blood vs PBMC: compartmental differences in gene expression profiling exemplified in asthma. Allergy Asthma Clin Immunol. 2019;15:67. 10.1186/s13223-019-0382-x 31832069 PMC6873413

[12] Riedhammer C , Halbritter D , Weissert R . Peripheral blood mononuclear cells: isolation, freezing, thawing, and culture. Methods Mol Biol. 2016;1304:53‐61. 10.1007/7651_2014_99 25092056

[13] Wang X , Bai H , Ma J , et al. Identification of distinct immune cell subsets associated with asymptomatic infection, disease severity, and viral persistence in COVID‐19 patients. Front Immunol. 2022;13:812514. 10.3389/fimmu.2022.812514 35281000 PMC8905648

[14] Nguyen NT , Chinn J , De Ferrante M , Kirby KA , Hohmann SF , Amin A . Male gender is a predictor of higher mortality in hospitalized adults with COVID‐19. PLoS One. 2021;16(7):e0254066. 10.1371/journal.pone.0254066 34242273 PMC8270145

[15] Yu C , Littleton S , Giroux NS , et al. Mucosal‐associated invariant T cell responses differ by sex in COVID‐19. Med (N Y). 2021;2(6):755‐772. 10.1016/j.medj.2021.04.008 33870241 PMC8043578

[16] Guo C , Li B , Ma H , et al. Single‐cell analysis of two severe COVID‐19 patients reveals a monocyte‐associated and tocilizumab‐responding cytokine storm. Nat Commun. 2020;11(1):3924. 10.1038/s41467-020-17834-w 32764665 PMC7413381

[17] Singh NK , Srivastava S , Zaveri L , et al. Host transcriptional response to SARS‐CoV‐2 infection in COVID‐19 patients. Clin Transl Med. 2021;11(9):e534. 10.1002/ctm2.534 34586723 PMC8453261

[18] Ng DL , Granados AC , Santos YA , et al. A diagnostic host response biosignature for COVID‐19 from RNA profiling of nasal swabs and blood. Sci Adv. 2021;7(6):eabe5984. 10.1126/sciadv.abe5984 PMC7857687 33536218

[19] Caniego‐Casas T , Martinez‐Garcia L , Alonso‐Riano M , et al. RNA SARS‐CoV‐2 persistence in the lung of severe COVID‐19 patients: a case series of autopsies. Front Microbiol. 2022;13:824967. 10.3389/fmicb.2022.824967 35173701 PMC8841799

[20] Delorey TM , Ziegler CGK , Heimberg G , et al. COVID‐19 tissue atlases reveal SARS‐CoV‐2 pathology and cellular targets. Nature. 2021;595(7865):107‐113. 10.1038/s41586-021-03570-8 33915569 PMC8919505

[21] Wu H , He P , Ren Y , et al. Postmortem high‐dimensional immune profiling of severe COVID‐19 patients reveals distinct patterns of immunosuppression and immunoactivation. Nat Commun. 2022;13(1):269. 10.1038/s41467-021-27723-5 35022412 PMC8755743

[22] Pujadas E , Beaumont M , Shah H , et al. Molecular profiling of coronavirus disease 2019 (COVID‐19) autopsies uncovers novel disease mechanisms. Am J Pathol. 2021;191(12):2064‐2071. 10.1016/j.ajpath.2021.08.009 34506752 PMC8423774

[23] Desai N , Neyaz A , Szabolcs A , et al. Temporal and spatial heterogeneity of host response to SARS‐CoV‐2 pulmonary infection. Nat Commun. 2020;11(1):6319. 10.1038/s41467-020-20139-7 33298930 PMC7725958

[24] Bost P , De Sanctis F , Cane S , et al. Deciphering the state of immune silence in fatal COVID‐19 patients. Nat Commun. 2021;12(1):1428. 10.1038/s41467-021-21702-6 33674591 PMC7935849

[25] Huang L , Li X , Gu X , et al. Health outcomes in people 2 years after surviving hospitalisation with COVID‐19: a longitudinal cohort study. Lancet Respir Med. 2022;S2213‐2600(22):00126‐6. 10.1016/S2213-2600(22)00126-6 PMC9094732 35568052

[26] Ghandikota S , Sharma M , Jegga AG . Computational workflow for functional characterization of COVID‐19 through secondary data analysis. STAR Protoc. 2021;2(4):100873. 10.1016/j.xpro.2021.100873 34746856 PMC8551262

[27] Cao Y , Xu X , Kitanovski S , et al. Comprehensive comparison of RNA‐Seq data of SARS‐CoV‐2, SARS‐CoV and MERS‐CoV infections: alternative entry routes and innate immune responses. Front Immunol. 2021;12:656433. 10.3389/fimmu.2021.656433 34122413 PMC8195239

[28] Jha PK , Vijay A , Halu A , Uchida S , Aikawa M . Gene expression profiling reveals the shared and distinct transcriptional signatures in human lung epithelial cells infected with SARS‐CoV‐2, MERS‐CoV, or SARS‐CoV: potential implications in cardiovascular complications of COVID‐19. Front Cardiovasc Med. 2020;7:623012. 10.3389/fcvm.2020.623012 33521069 PMC7844200

[29] Cavalli E , Petralia MC , Basile MS , et al. Transcriptomic analysis of COVID19 lungs and bronchoalveolar lavage fluid samples reveals predominant B cell activation responses to infection. Int J Mol Med. 2020;46(4):1266‐1273. 10.3892/ijmm.2020.4702 32945352 PMC7447313

